# Patent Landscape of Fiber-Based Fabrication Technologies for Functional Biomaterials: Electrospinning, Forcespinning^®^ and Melt Electrowriting in Tissue Engineering and Drug Delivery (2020 to 2024)

**DOI:** 10.3390/jfb17010008

**Published:** 2025-12-22

**Authors:** Amelie Maja Sattler, Marisela Rodriguez-Salvador, Javier Vazquez-Armendariz, Raquel Tejeda Alejandre

**Affiliations:** 1 School of Engineering and Sciences, Tecnologico de Monterrey, Monterrey 64700, Mexico; amelie_sattler@gmx.de (A.M.S.); vazquezarmendariz.1@osu.edu (J.V.-A.); raquel.tejeda@tec.mx (R.T.A.); 2 Department of Integrated Systems Engineering, The Ohio State University, Columbus, OH 43210, USA

**Keywords:** electrospinning, melt electrowriting, Forcespinning^®^, tissue engineering, drug delivery, competitive technology intelligence, patents

## Abstract

Electrospinning, Forcespinning^®^, and melt electrowriting are becoming increasingly important fiber-based fabrication technologies for tissue engineering and drug delivery applications. Despite their scientific and industrial relevance, their patent landscape has not been systematically examined, which limits the understanding of technological dynamics and translational applications. This study addresses this gap through a patentometric analysis conducted within a Competitive Technology Intelligence framework. A total of 3557 active and granted Extended Patent Families from 2020 to 2024 were analyzed to identify temporal patterns, geographic distribution, key innovators, industrial sectors, and primary application areas. The results showed that the overall patent activity increased until 2022 before experiencing a slight decline. China dominates the landscape, accounting for approximately 62% of applications filed, largely driven by academic institutions such as Shanghai University. Leading industries include special-purpose machinery, medical and dental technology, and textiles. According to International Patent Classification codes, filament formation (D01D5/00) is prevalent, while electrospinning—specifically IPC D04H1/728—represents the most active and influential of the three technologies. These findings exhibit the technological dynamics shaping fiber-based fabrication platforms and underscore their growing relevance in pharmaceutical innovation. The identified trends position these technologies as foundational for next-generation biomaterial design, offering valuable insights for researchers, industry stakeholders, and policymakers.

## 1. Introduction

Fiber-based fabrication processes such as electrospinning, Forcespinning^®^, and melt electrowriting are gaining increasing interest in both the scientific community and industry [1,2,3]. These techniques enable the production of ultrafine polymeric fibers that exhibit remarkable characteristics, including high permeability, porosity, stability, and flexibility, as well as a large surface-to-volume ratio and tunable mechanical properties [4,5,6,7]. Owing to these distinctive features, the fabricated fibers are being explored for a wide range of applications across multiple fields, including energy, environmental engineering, biotechnology, and biomedicine [8,9,10]. Notably, they have been employed in wound dressings [11], drug delivery systems [12,13], tissue engineering scaffolds [14,15,16], filtration media [17], biosensors [18,19], and protective clothing [4,20,21,22].

In particular, such attributes make them ideal platforms for creating functional biomaterials, engineered to interact with biological systems and deliver therapeutic or regenerative outcomes. For pharmaceutical purposes, these fiber-based fabrication technologies offer unique advantages, as they enable the design of advanced drug delivery systems and pharmaceutical dosage forms with enhanced functionality [4]. To date, the vast majority of research on drug encapsulation in fibers has focused on electrospinning for various pharmacological applications, including antibiotic, cardiovascular, ocular, oral, contraceptive, and antihistamine treatments with instant, extended, or controlled release [22]. The ability to control fiber size, morphology, composition, and architecture at the micro- and nanoscale allows for precise modulation of drug loading, release kinetics, and bioavailability, which are critical parameters in developing effective therapies. Moreover, the high surface area and porosity of electrospun and melt electrowritten fibers may facilitate the rapid dissolution of poorly soluble drugs [5,6]. In contrast, multilayered or composite fiber constructs can be engineered for sustained or targeted release of therapeutic agents. Together, these features underscore electrospinning, Forcespinning^®^, and melt electrowriting as powerful technologies for engineering functional biomaterials that address critical challenges in patient compliance, controlled drug administration, and site-specific therapeutic action [12,23].

### 1.1. Electrospinning

**Figure 1:**
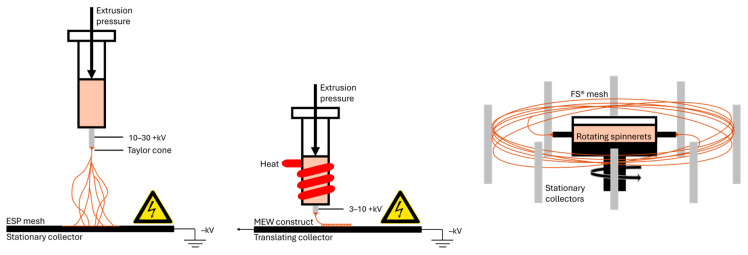
From left to right, schematic representation of the electrospinning, melt electrowriting and Forcespinning^®^ set up. Please note that the figures are just representative and do not resemble the real size of the setup.

The electrospinning process involves preparing a polymer solution in a reservoir, typically a syringe with a needle that serves as a charged nozzle. A high voltage is then applied to create an electric field between the tip of the nozzle and the electrically grounded collector. Subsequently, a thin jet of the polymer solution is ejected from the nozzle tip towards the collector. As a result, solid fibers are collected into a mesh in a random alignment [14]. Typical solution-based electrospinning can produce fiber sizes in the range of 20 nm to 10+ µm when optimizing key parameters, such as the viscosity of the solution, voltage, and feed rate [17]. To modulate the mechanical, chemical, and functional properties of fiber meshes for different applications, various electrospinning setups have been reported, including coaxial, triaxial, side-by-side, and multichannel [18]. It is also possible to modify these properties during or after the fiber generation by using crosslinking strategies, which is referred to as reactive electrospinning [20]. Please refer to Figure 1 to observe a schematic representation of an electrospinning setup.

### 1.2. Forcespinning^®^

Forcespinning^®^, also known as rotor spinning, centrifugal jet spinning, or centrifugal spinning, is an alternative technique for fabricating nanofibers. The registered term was popularized by the company FibeRio^®^ Technology Corporation (McAllen, TX, USA), which patented and marketed this technology until 2016 before being acquired by an undisclosed party [24]. In this technique, the polymer solution or melt is expelled from a rotating spinneret, where centrifugal force overcomes the surface tension of the liquid, initiating a stretching process that transforms the solution or molten material into fibrous structures [25] (see Figure 1). Unlike electrospinning, this technique does not require the use of high-voltage electrical fields [21]. Forcespinning^®^ can generate fibers ranging from 250 nm to 20+ µm by tuning the polymer concentration of the solution, spinning speed, and nozzle size [21]. The versatility of this technique is reflected in its capacity to process a wide range of materials, encompassing both synthetic and natural polymers, to yield fibers with controlled morphologies suitable for advanced applications [22,26]. Please refer to Figure 1 to observe a schematic representation of a Forcespinning^®^ setup.

### 1.3. Melt Electrowriting

The fundamental principles behind this process combine electrospinning and melt-extrusion-based techniques, as the electrostatic forces created by an electrical field applied between the nozzle and a translating collector platform produce the stretching of the molten polymer jet into fibers, creating a tunable microarchitecture in a three-dimensional space [27,28]. In contrast to electrospinning, solvents are rarely used in the production of melt electrowriting fibers. By optimizing printing parameters such as the Critical Translation Speed (CTS) of the collector, high voltage, and extrusion rate, highly aligned fibers in the range of 5–50 µm can be deposited via melt electrowriting [29]. The control of jet stability, which also influences the fiber trajectory and deposition accuracy, is known to be affected by electrostatic forces when attempting to print on out-of-plane substrates [30]. Please refer to Figure 1 to observe a schematic representation of a melt electrowriting setup.

### 1.4. Research Importance and Focus

Despite substantial progress in understanding electrospinning, Forcespinning^®^, and melt electrowriting individually, a consolidated patent analysis that captures their collective technological evolution has been notably absent [27,28,31]. Addressing this is essential, as patent data provide a unique lens into innovation trajectories, technological maturity, and competitive positioning—dimensions not fully accessible through traditional scientific literature. To fill this gap, a Competitive Technology Intelligence (CTI) methodology was applied, focusing on a patentometric analysis to determine the technological landscape of the three fiber-based technologies for tissue engineering and drug delivery: electrospinning, Forcespinning^®^, and melt electrowriting. A comprehensive biomedical assessment would ideally integrate patents with experimental, clinical, and industrial data. However, the present work is intentionally positioned within the field of patentometrics, which focuses on analyzing innovation trends, technological development, and knowledge flows as reflected in patent documents. Patentometric studies do not aim to replace experimental or clinical validation but rather to provide a complementary, systematic overview of technological evolution in a given domain. Such a study can offer valuable insights for the biomaterials community. By revealing temporal trends in patenting activity, technological dynamics, patent geographical distribution, leading innovators, industrial sectors, and patent principal application domains, this research aims to provide actionable insights that can guide and strategically orient research and innovation efforts in the development of functional biomaterials.

CTI is a systematic approach that is based on monitoring technical and competitive environments [32]. It involves a continuous process that facilitates both strategic planning and the identification of innovative developments [33,34] to support decision-making processes in the areas of technology innovation, technology forecasting, product design, and research and development (R&D) [32]. The CTI contributes to research by serving not only as a source of information but also by enabling the early identification of technological developments and their trends. This is particularly important in the fields of tissue engineering and drug delivery, where advances in fiber-based functional biomaterials are rapidly evolving to optimize therapeutic performance and regenerative outcomes. In summary, CTI can provide a forward-looking framework for anticipating technological changes and decision-making for the development of future biomaterials.

The study shows how patent activity mirrors technological advancements in the development of sophisticated systems engineered to interface with biological environments and enhance therapeutic outcomes.

## 2. Methodology

**Figure 2:**
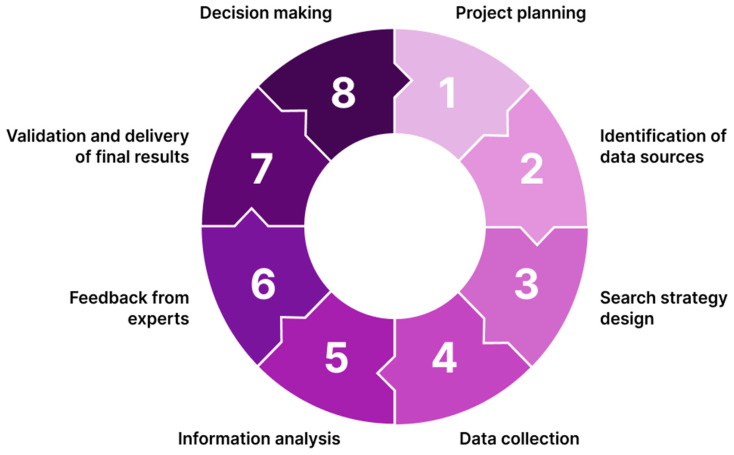
Competitive technology intelligence (CTI) eight-step methodology, Adapted from Ref. [35].

This study employed the CTI methodology developed by Rodriguez-Salvador and Castillo-Valdez in 2021 [35], which is based on an eight-step process involving interrelated stages and ongoing feedback. That includes: (1) project planning, (2) identification of data sources, (3) search strategy design, (4) data collection, (5) information analysis, (6) feedback from experts, (7) validation and delivery of final results, and (8) decision making (Figure 2).

The first step involved planning the organization of tasks to accomplish the main project’s goal: to determine temporal trends in patenting activity, its geographical distribution, key players, industry sectors, and application domains in electrospinning, Forcespinning^®^, and melt electrowriting technologies for tissue engineering and drug delivery applications.

In the second step, primary and secondary sources were identified. The primary sources included industry and academic experts in the fiber-based fabrication technologies analyzed, who were consulted at various stages of the methodology. Secondary sources included patents extracted from PatSeer, a patent software platform with access to more than 172 million patents worldwide [36]. It covers patent data from more than 100 patent authorities, including full-text records from major jurisdictions such as the United States of America, Europe, China, and Japan. And it is certified by the International Standards Organization (ISO) [36].

**Figure 3:**
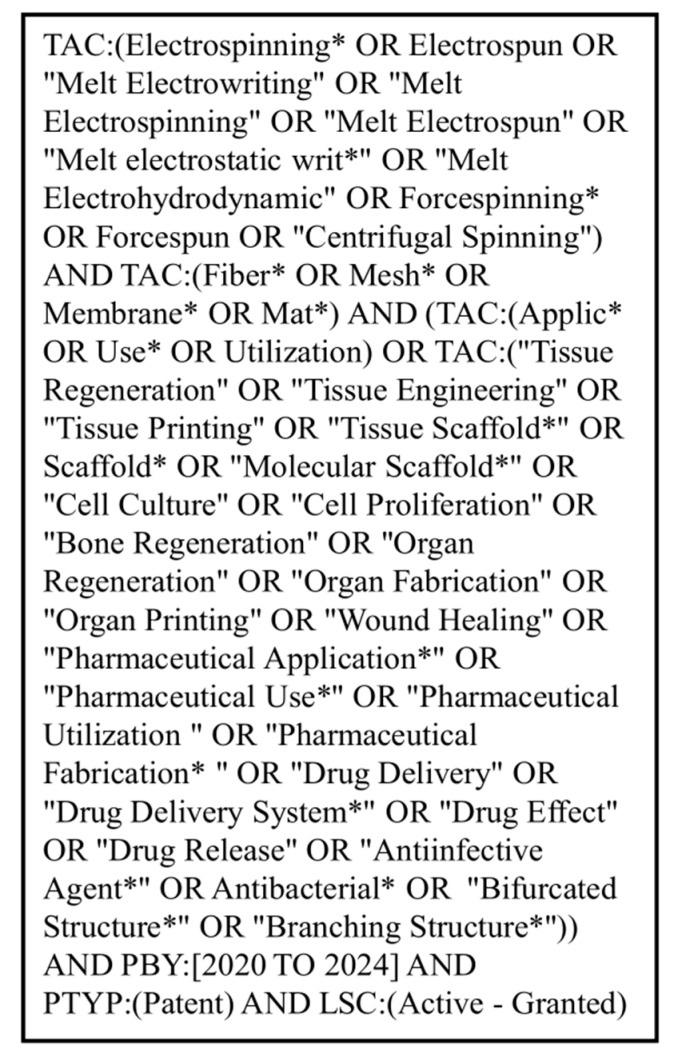
Patent search query used in this study. It covered patents from 2020 to 2024 for electrospinning, melt electrowriting, and Forcespinning^®^ technologies for tissue engineering and drug delivery applications.

Step three focused on developing the search strategy. To ensure proper patent collection, the experts involved in this study identified and validated the relevant key terms and results. Based on the resulting terminology, a search query was developed using synonyms and variations in the key terms to match the objective. The analysis period was set as patents published between 2020 and 2024. Boolean operators such as AND, OR, NOT were applied iteratively, with different queries tested to develop the best approach, aiming to ensure relevant data was identified and extracted. Figure 3 illustrates the final query created.

For the fourth step, data were collected using Extended Patent Family (EFAM) search criteria. According to the European Patent Office (EPO), an Extended Patent Family refers to a collection of patent documents that share a common priority, meaning they have at least one priority application in common, either directly or indirectly [37]. This criterion enables more comprehensive coverage of all patents related to an invention, regardless of national or international property rights or variations in the original application. In contrast to the Simple Patent Family (SFAM), which considers only patents with identical technical content, EFAM was chosen to capture technological developments and their global protection strategies more precisely. Another critical aspect of data collection was the restriction on active and granted patents. This means that only patents that were both legally granted and in force at the time of the analysis were considered, excluding those that died, were withdrawn, or lapsed. This restriction was applied to ensure that the study focused on patents with current legal validity, which are more relevant for analyzing the state of technology. To ensure the quality and comparability of the information, the data were then normalized and pre-processed. This step was performed to confirm the accuracy, internal consistency, and contextual relevance of the data. The resulting database provided a robust basis for the subsequent analysis.

In the fifth step, the EFAM were analyzed to identify key players, patent dynamics, and to classify the patents into relevant technological fields. The experts participating in this study assessed the results obtained from the early stages to ensure accuracy. This activity was developed in the sixth step under an iterative approach, where adjustments were made accordingly until arriving at step seven, with the final results validation. The main insights were presented in Section 3 and Section 4. Finally, decision-making would take place in step eight, which oversees people involved in R&D&I activities in the field of study.

## 3. Results

By applying the process explained in Section 2 and the search query shown in Figure 3, a total of 3557 EFAMs were obtained from the PatSeer platform as of 21 April 2025. These patents were analyzed to uncover technological development dynamics across electrospinning, Forcespinning^®^, and melt electrowriting in the next sections. Beyond providing a quantitative overview of patent activity, this dataset offers a valuable lens for examining how innovative trajectories in fiber-based fabrication technologies advance the field, including functional biomaterials.

### 3.1. Patent Trends in Fiber-Based Fabrication Technologies for Tissue Engineering and Drug Delivery

**Figure 4:**
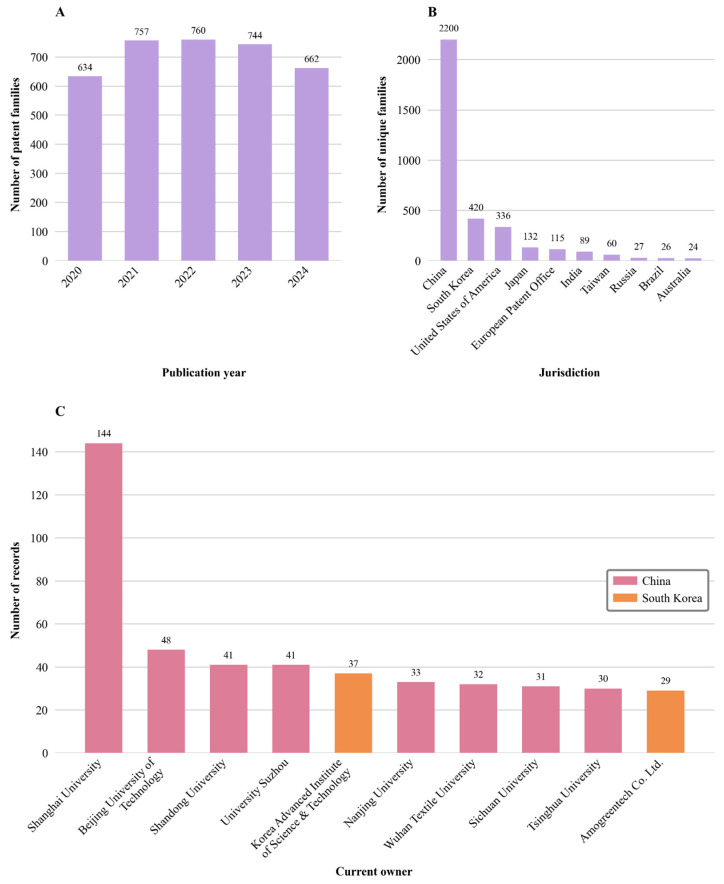
Global patenting trends for electrospinning, Forcespinning^®^, and melt electrowriting technologies for tissue engineering and drug delivery (2020–2024), including (**A**) the annual number of patent families by publication year; (**B**) the geographic distribution of unique families based on the jurisdiction of first filing; and (**C**) the distribution of patents by current owner.

The temporal development and global distribution of patent activity for all three fiber-based fabrication technologies for tissue engineering and drug delivery were determined with the help of PatSeer platform. Figure 4 provides a detailed overview of patenting trends from 2020 to 2024, including the annual number of published patent families, the jurisdiction of the first filings, and the current ownership patterns. By tracing the emergence of electrospinning, Forcespinning^®^, and melt electrowriting patents, including their location and timing, and identifying who holds them, it becomes possible to understand how global research and industrial ecosystems are shaping the development of advanced technologies, such as biomaterial platforms, with direct implications for regenerative medicine and drug delivery.

#### 3.1.1. Time-Based Dynamics of Patents

As shown in Figure 4A, in 2020, 634 patent families were published, whereas in 2021, this number increased to 757, representing a 19.4% rise. The peak of the observation period was 2022, when 760 patent families were published. However, by 2023, there was a slight decrease to 744, exhibiting a 2.1% reduction. This trend continued in 2024, with 662 patent families being published.

Beyond illustrating temporal fluctuations, these trends reflect shifts in research priorities, funding landscapes, and industrial interest in fiber-based fabrication technologies. The initial rise suggests rapid technological maturation and diversification of applications, while the slight decline may indicate consolidation as leading players strengthen their intellectual property portfolios. Importantly, sustained activity across the entire period confirms the long-term relevance of these technologies for the development of functional biomaterials, particularly in tissue engineering and drug delivery, where continuous innovation is essential to address therapeutic challenges.

#### 3.1.2. Patent Regional Distribution

While the previous section provides insights into the patent trajectory of the three fiber-based technologies analyzed, examining the geographic distribution of filings offers complementary insights into the broader invention landscape explored in this study. Specifically, observing the jurisdictions of the first filing can provide critical insight into regionally concentrated invention activities. Therefore, as part of this research, an analysis of the jurisdictions where the patents were granted was executed to identify the dynamics of patents issued by each patent authority. In the context of patent law, jurisdiction refers to the legal authority granted to a national or regional patent office to examine, grant, and enforce patents within a specific geographic area [38]. Each jurisdiction represents a specific territory, with its own legal and institutional framework for intellectual property protection.

To ensure greater comparability and avoid distortions caused by multiple filings of the same invention, the geographical analysis performed was focused on the concept of “unique families”. Each invention was counted only once, regardless of the number of individual filings by the different authorities. The jurisdiction of each patent was determined by the country of the first filing, thereby enabling a more accurate attribution of the invention’s origin. This methodological choice forms the basis for the geographical assessment presented in Figure 4B, covering the period 2020 to 2024.

As shown in this Figure, the analysis of unique family publications reveals a notable, yet not geographically concentrated, distribution of inventions. Findings indicate that invention activities are primarily focused on Asia, particularly in China. From the 3557 unique families detected, this country registered 2200 unique families, which account for 61.84% of all publications and therefore represent the largest jurisdiction. China filed more than five times as many unique families as the second position held by South Korea, which registered 420 unique families. South Korea is ahead of the United States of America (USA), which published 336 unique families. In the following place, Japan has 132 unique families. The European Patent Office (EPO) ranks fifth, with 115 unique families, followed by India, which ranks sixth with 89 unique families. Taiwan and Russia had 60 and 26 unique families, respectively. Brazil and Australia complete the top 10 with 26 and 24 unique families, respectively. While Asia leads in patenting activity for fiber-based fabrication technologies, the contributions from European and North American institutions, as evidenced by filings from the EPO and the USA, highlight that industrialized Western countries also remain actively engaged in their development and application.

In summary, research and development of fiber-based fabrication technologies are recognized globally. Although Asian countries currently lead in patenting activity, the findings highlight the international significance of fiber-based fabrication technologies, as their applications in different areas, such as tissue engineering and drug delivery, are being actively explored across diverse regions and research ecosystems.

#### 3.1.3. Patent Ownership Landscape

Following the analysis of the geographic distribution of patent filings, an examination of ownership was conducted. This perspective allows for the identification of key players and reveals patterns of regional concentration that complement the global trends observed in jurisdictional patent activity. By highlighting the entities driving innovation in fiber-based fabrication technologies, this study offers insights into how leadership in this space translates into the development of different applications, including functional biomaterials. Since these materials can facilitate interaction with biological systems to achieve therapeutic or regenerative effects, understanding who controls the intellectual property is critical for anticipating future advances in tissue engineering, drug delivery, and related biomedical applications.

The analysis performed was based on individual patents rather than on patent families. This enables a more precise representation of ownership structures and avoids the aggregation effects that result from counting at the family level. Figure 4C shows the top 10 current patent owners. Similarly to Figure 4B, there is a clear concentration of patents in China, which demonstrates its dominance in the geographic distribution of first filings. The data are based on the number of patents assigned to the patent organizations of current owners as of 21 April 2025, which offers insights into institutional invention dynamics. By analyzing this information, additional insights can be gained into the research priorities that drive the invention of fiber-based fabrication technologies. In particular, the concentration of current ownership among a few institutions reflects structural patterns in the organization of invention activities.

Chinese institutions lead the patent ranking, with Shanghai University holding 144 patents, followed by Beijing University of Technology with 48 patents, and then the Universities of Shandong and Suzhou, each with 41 patents. The Korean Advanced Institute of Science and Technology (KAIST), based in South Korea, ranks fifth with 37 patents. Other notable Chinese institutions include Nanjing University, with 33 patents; Wuhan Textile University, with 32; Sichuan University, with 31; and Tsinghua University, with 30, ranking ninth.

Notably, Amogreentech Co., Ltd., based in South Korea, occupies the tenth position in the patent ranking, with 29 patents, making it the only private company on this list. The geographical distribution of the current owners shows a clear regional concentration in the Chinese economic centers of Shanghai, Beijing, and Shandong.

In summary, the analysis highlights the dominant role of Chinese research institutions in the fiber-based technology sector, with Shanghai University emerging as the leading actor. This leadership reflects China’s rapidly expanding innovation capacity, which may drive the development of advanced drug delivery systems and pharmaceutical formulations, particularly through scalable fiber production and nanotechnology integration. Notably, in terms of technologies, China has placed significant emphasis on patenting electrospinning technologies, although not all of these inventions have been commercialized. In contrast, while the number of Chinese patents in melt electrowriting is lower, a greater proportion of these innovations have successfully reached the market.

Finally, the presence of the Korea Advanced Institute of Science & Technology (KAIST) and Amogreentech Co., Ltd. (Kr) shows that international academic institutions and private sector companies also contribute to inventions in the field of fiber-based fabrication technologies.

### 3.2. Patent Landscape for Emerging Fiber Fabrication Technologies

To identify which sectors benefit most from advances in fiber-based fabrication technology, an analysis was conducted of the ten leading industries ranked by the number of unique patent families. This approach provides valuable insights into how progress in electrospinning, Forcespinning^®^, and melt electrowriting is being translated into practical applications. By revealing the sectors most actively investing in patent protection, this analysis underscores the pathways through which fiber-based fabrication contributes to the development of new solutions.

**Figure 5:**
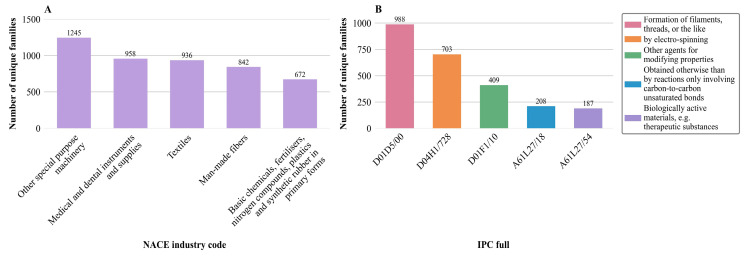
Patent landscape for electrospinning, melt electrowriting, and Forcespinning^®^ technologies for tissue engineering and drug delivery (2020–2024) based on unique family analysis. (**A**) The top five Statistical Classification of Economic Activities in the European Community (NACE) industry codes and (**B**) the top five International Patent Classification (IPC) full classes.

In this context, the analysis considered the Statistical Classification of Economic Activities in the European Community (NACE) Industry codes. NACE is a standardized classification system that has been used in the European Union since 1970 to categorize economic activities systematically [39]. It enables consistent collection and analysis of economic data, allowing a comparable view in areas such as production, employment, and innovation. As shown in Figure 5A, the chart does not assign each patent family to one industry. Instead, all industries associated with any patent within a family are considered. Each family is therefore included once per relevant industry, which is referred to as a “unique family”. This broader perspective enables improved insights into the cross-sectoral impact and technological reach of fiber-based fabrication developments, thereby enhancing the interpretability and strategic value of the analysis.

The NACE code “Other special purpose machinery” has the highest invention activity, containing 1245 unique families, where specialized machinery is particularly notable. The second position, with 958 unique families, is related to “Medical and dental instruments and supplies”, which underscores the high relevance of these technologies for the health industry, particularly in areas such as tissue engineering and drug delivery. The “Textiles” industry is almost equally important, with 936 unique families, followed by “Man-made fibers” with 842. These figures reflect the fact that the development of synthetic fibers through processes such as Forcespinning^®^, melt electrowriting, and electrospinning is a driving force for inventions. In fifth place with 672 unique families is “Basic chemicals, fertilizers, nitrogen compounds, plastics, and synthetic rubber in primary forms”. The proximity of the number of unique families of the codes “Medical and dental instruments and supplies”, “Textiles”, and “Man-made fibers” indicates a strong link between material science and application-oriented technologies.

Collectively, the results indicate a high concentration of patents in technology-intensive areas, including specialized machinery, medical technology, textile production, and the chemical processing industries. This highlights the interdisciplinary significance of fiber-based manufacturing technologies and their increasing importance in innovative applications, including tissue engineering and drug delivery. In particular, the convergence of technology-intensive fields in drug delivery and pharmaceutical formulation facilitates the development of multifunctional fiber platforms capable of precise drug encapsulation, controlled release, and improved bioavailability [6,12]. The incorporation of chemical processing expertise supports the scalable production of fibers with tailored physicochemical properties, while advances in medical technology enable their integration into therapeutic devices and dosage forms. This interdisciplinary overlap not only accelerates the design of innovative pharmaceutical strategies but also strengthens the path from laboratory-scale fiber fabrication towards the application of clinically relevant drug delivery systems [4].

While this analysis, based on the NACE classification, underscores the broad industrial relevance of fiber-based fabrication technologies, a more detailed perspective can be obtained through a complementary examination using the International Patent Classification. Unlike sector-based classifications, the IPC provides a systematic categorization of the underlying technical principles and specific application contexts. This perspective enables the identification of patent activity directly related to pharmaceutical applications.

The International Patent Classification (IPC) is a system designed to classify inventions and facilitate the evaluation of their characteristics systematically [40]. By evaluating the IPC codes, it is possible to identify the main technological focuses in a particular field of invention. In this section, the top five IPC full codes, shown in Figure 5B, were determined based on the number of patent families of fiber-based fabrication technologies, in particular, electrospinning, Forcespinning^®^, and melt electrowriting, which are the focus of this study. In this context, “full” refers to the complete set of IPC classifications, including both the primary and any secondary codes, that have been assigned to all patent documents within a family. It is important to note that the chart does not rely on a single representative record per patent family. Instead, all IPC full classes assigned to any member of each family are considered. Each distinct family is counted once, regardless of how many of its documents carry the same classification, resulting in what is referred to as a “unique family”. This approach provides a more comprehensive representation of the technological scope associated with each family.

The analysis of the data reveals that the IPC code D01D5/00, “Formation of filaments, threads, or the like,” dominates with 988 unique families. This underlines the central role of fiber production technologies, which extend beyond traditional textile manufacturing into advanced fields such as materials science and biomedical engineering. These processes are essential for producing a wide range of structural forms, including woven and nonwoven fabrics, as well as high-performance materials for various applications. When applied to the biomedical domain, they enable the fabrication of scaffolds and matrices that serve as functional biomaterials, supporting cell growth, tissue regeneration, and controlled drug delivery. Therefore, the predominance of this IPC category highlights not only the versatility of fiber-based technologies but also their strategic importance in shaping next-generation therapeutic and regenerative solutions.

The second principal code, D04H1/728 “by electrospinning”, is represented by 703 unique families and highlights the relevance of nanofiber production, particularly for medical applications such as tissue engineering and controlled drug release. It follows the IPC code D01F1/10 “Other agents for modifying properties” represented by 409 unique families. This highlights the importance of optimizing material properties, which is crucial for developing application-oriented, functionalized fiber materials. In fourth position is the IPC full code A61L27/18 “obtained otherwise than by reactions only involving carbon-to-carbon unsaturated bonds” with 208 unique families. This code refers to materials produced using alternative chemical processes that do not rely exclusively on reactions between carbon-carbon double bonds. This underlines the growing interest in synthesis methods for creating functional biomaterials that meet specific requirements, such as biocompatibility or controlled degradation behavior. And ranked as fifth is the IPC code A61L27/54 “Biologically active materials, e.g., therapeutic substances” with 187 unique families, highlighting the growing importance of biologically active materials that contain, for example, therapeutic agents or trigger targeted biological reactions in the body. Such materials are of particular interest for applications in controlled drug release, regenerative medicine, and implant technology.

In summary, the construction of filaments, threads, or similar products, and the use of electrospinning are the principal categories according to the IPC analysis. The dominance of these categories suggests that a substantial proportion of innovation is centered on methods that can be directly applied in the medical field, such as creating advanced drug delivery systems, scaffolds for regenerative medicine, and multifunctional pharmaceutical materials. The manufacturing of filaments and threads provides versatile platforms for pharmaceutics, enabling the development of drug-loaded scaffolds, sutures, and implantable systems with controlled release profiles.

The rationale behind the prevalence of electrospinning over Forcespinning^®^, and melt electrowriting stems from several factors. Historically, the electrospinning technique was popularized in the 1990s for the fabrication of fibrous meshes for various applications, and since then, many related patents have been issued [41]. Our findings indicate that electrospinning stands out as the dominant technique, owing to its unique ability to produce nanofibers with very high surface-area-to-volume ratios and tunable morphologies, which, for pharmaceutical applications, facilitate improved drug solubility, bioavailability, and targeted delivery [42,43,44,45]. Electrospinning has proven particularly advantageous for incorporating Biopharmaceutics Classification System (BCS) Class II and Class IV drugs, as well as heat-sensitive biomolecules such as proteins and enzymes. Because the process does not require elevated temperatures, it helps preserve the integrity of thermolabile compounds while enhancing the solubility, dissolution rate, bioavailability, and overall stability of poorly water-soluble APIs. By strengthening the ability of sensitive biomolecules, it favors stability and optimizes performance, capabilities that are progressively improved by advances in polymer design and precise control over fiber architecture [5,43,46,47]. This makes electrospinning a versatile and scalable platform for formulating challenging therapeutic agents [42]. In this context, electrospun polymeric fibers are of particular interest in the biomaterials industry due to their distinctive properties. For example, their high surface-to-volume ratio and interconnected porosity facilitate efficient cell adhesion, nutrient diffusion, and tissue growth [48]. Moreover, the fibrous morphology of electrospun meshes to conform tissue engineering scaffolds closely emulates the architecture of the native extracellular matrix, providing an appropriate microenvironment for cellular proliferation and differentiation [14]. The tunable morphology, composition, and mechanical performance of electrospun fibers, together with their amenability to surface functionalization and therapeutic loading, further enhance their potential for diverse biomedical applications [21,22].

The scientific principles of Forcespinning^®^ and melt electrowriting were introduced for the first time between 2010 and 2011 and can therefore still be considered techniques under development [49]. Forcespinning offers a high-throughput, solvent-compatible route to produce micro- and nanofibers with architectures relevant to tissue engineering. Studies comparing electrospinning and rotary-jet spinning show that both can generate biocompatible poly(ε-caprolactone) (PCL) scaffolds. Still, Forcespun fibers exhibit distinct surface roughness that supports cell adhesion while markedly reducing bacterial colonization, without the need for antimicrobial additives. This topography-driven effect underscores the value of Forcespinning for creating cytocompatible, structurally tunable scaffolds with inherent functional advantages [48]. On the other hand, Melt electrowriting (MEW), which is an emerging solvent-free additive manufacturing technique, can enable highly controlled microarchitectures, making it particularly promising for scaffold fabrication. Functionalized MEW-fabricated 3D architectures are increasingly employed to emulate key features of the extracellular matrix, offering a provisional yet structurally defined environment that supports organized three-dimensional tissue growth and maturation [50]. Its ability to produce non-linear fiber geometries offers enhanced structural and mechanical biomimicry; however, achieving accurate deposition of complex patterns remains challenging due to jet lag and discrepancies between the programmed toolpath and the deposited fibers. Recent efforts have increasingly focused on improving the precision of non-linear fiber deposition in MEW to reduce the gap between intended and actual printed scaffold architectures [51]. A recent study demonstrated that MEW could achieve micrometric precision in fabricating fibrous aortic root scaffolds with anatomically accurate geometries, biomimetic microstructures, and mechanical properties [51]. The same work further showed that MEW can generate fully patient-specific aortic root constructs derived directly from computed tomography-based 3D reconstructions, underscoring its potential for personalized soft-tissue biomanufacturing. In parallel, studies examining biomedically relevant polymers processed via Near-field electrospinning (NFES) and MEW have been developed, and ongoing limitations in direct fiber writing have also been analyzed, including insufficient standardization of setup parameters, constrained fiber-writing throughput, and technical barriers to reliably producing complex scaffold geometries [52]. Together, these challenges underscore the need for coordinated advances in material selection, process control, and reporting practices to fully leverage MEW for high-fidelity biomedical scaffold fabrication.

Cost and accessibility have also played an essential role in the dominance of electrospinning. In a standard electrospinning setup, the most significant investment is the cost of acquiring the high-voltage source and the feeding solution system. Other components can be added to increase the technique’s versatility, but in general, the standard setup can be easily replicated [18]. Melt electrowriting requires a precise translation platform for fiber collection and a heating system for the polymer [29]. Similarly, Forcespinning^®^ also requires an electromechanical system for spinning of the nozzle and a series of stationary collector plates for fiber collection [53]. Although it is possible to replicate the setup for both techniques, a larger investment is required, which can hinder their accessibility.

### 3.3. Most Cited Patent Families in Fiber-Based Fabrication Technologies

Patents with high citation counts often serve as critical foundations for subsequent research and innovations, shaping technological trajectories across multiple domains [54]. In the context of fiber-based fabrication technologies, highly cited patents emphasize seminal contributions that have established key methods or applications, influencing the evolution of electrospinning, Forcespinning^®^, and melt electrowriting within both academic and industrial settings. Examining the most-cited patent families yields valuable insights into the technological pillars driving innovation and helps identify breakthroughs most relevant to future progress in biomedical applications. Table 1 provides the top five most cited patents in fiber-based technologies according to our analysis developed in Patseer platform, highlighting their influence on scientific and industrial developments.

This analysis was based on data retrieved on 21 April 2025 and covers patents published between 2020 and 2024. The results showed that the number of citations in the top five ranged from 51 to 113.

The most cited patent, with 113 citations, is US10849754B2, titled “Heart valve sealing devices and delivery devices therefor,” published in 2020 and currently owned by the Edward Lifesciences Corporation and based in Irvine, CA, USA. This report describes a minimally invasive repair device designed for the treatment of native valve insufficiency. Its core structure consists of a hollow coaptation element designed to facilitate valve leaflet approximation and a paddle system actuated by a central shaft mechanism. A notable feature is the use of electrospun textiles for a coaptation structure, which promotes flexibility, tissue conformity, and tissue integration. This result illustrates how fiber-based fabrication technologies are enabling the development of advanced biomedical solutions that rely on functional biomaterials. Although this invention is not directly focused on drug delivery, its impact lies in the design of a minimally invasive implantable device that integrates material science and engineering principles to interact with biological tissues therapeutically. The coaptation element and paddle system exemplify how carefully engineered materials can restore function to diseased organs.

The US11013600B2 “Covered prosthetic heart valve” published in 2021, is ranked second with 109 citations and is also owned by the Edward Lifesciences Corporation. This patent introduces a prosthetic valve with a radially collapsible stent frame and a multi-layered textile covering. The cover incorporates texturized polyethylene terephthalate (PET) strands and low-friction electrospun layers, designed to support radial expansion of the stent structure while minimizing shear forces during deployment. From a biomaterials perspective, this invention exemplifies the integration of fiber-based fabrication technologies into the design of implantable devices that must operate reliably with the dynamic biological system of the heart. The use of electrospun fibers highlights the importance of tailoring surface properties and mechanical behavior to achieve biocompatibility, stability, and functional performance, making it a compelling example of how functional biomaterials are central to medical device innovations.

The third most cited patent, with 66 citations, is US10822542B2, “Perovskite/polymer composite luminescent material,” published in 2020 and currently owned by the Beijing Institute of Technology and Zhijing Nanotech. This patent introduces a novel fabrication method in the domain of electrospun nanocomposites. The patent reports a fabrication process in which perovskite nanoparticles are embedded within polymeric matrices, such as polyvinylidene fluoride (PVDF) and cellulose acetate (CA), via electrospinning. The resulting fibrous films exhibit mechanical flexibility, structural porosity, and tunable optical properties. Although initially developed for optoelectronic applications, the material configuration closely parallels that of bioactive scaffolds used in tissue engineering and nanostructured carriers for drug delivery. Thus, this patent reveals a translational material platform at the interface of photonics and biomedicine, demonstrating how functionalized fibers can support emerging applications in biosensing, responsive drug release, and implantable diagnostic systems.

The fourth place with 58 citations is US10993809B2, also titled “Heart valve sealing devices and delivery devices therefor” and is likewise currently owned by the Edward Lifesciences Corporation. It involves a sealing and stabilization system for heart valve prostheses from a single woven strip. This woven strip is composed of electrospun polymeric or metallic fibers, enabling minimally invasive transcatheter access.

Ranked fifth is US 11167058 B2, “Hemostasis of wound having high pressure blood flow,” with 51 citations, currently owned by the Virginia Commonwealth University in the USA. This patent introduces an innovative method for achieving hemostasis in wounds characterized by high blood pressure, utilizing electrospinning to incorporate hemostatic agents such as kaolin into fiber-based bandages. This technology addresses challenges associated with blood clotting in injuries with heavy blood flow, commonly occurring from arterial bleeding or a location of intentional trauma. The innovation of this patent lies in the development of a new system that can effectively stop blood flow without relying on conventional invasive methods, which are often associated with significant risks and complications. This invention demonstrates how fiber-based fabrication can be optimized to produce structures that combine mechanical strength, flexibility, and biocompatibility, essential properties for safe and effective cardiovascular implants.

Table 1 also provides deeper insight into the key innovations and materials used in the top five most-cited patents. These highly cited patents underscore the crucial role of fiber-based fabrication methods in driving technological advancements across various industries, including medical technology and related fields. Their influence is likely to shape future developments and inventions in these sectors. Notably, most patents are currently assigned to US-based institutions, with three of these held by Edwards Lifesciences Corporation, reflecting a highly consolidated innovation strategy in the medical device sector. Electrospinning was mentioned in all five of the most cited patents, indicating that this fabrication method was the most impactful among the fiber-based technologies under examination. The dominance of electrospinning underscores its importance and versatile applicability in a range of advanced materials for medical applications.

Taken together, the analysis of the five most-cited patents reveals several emerging trends that characterize the current trajectory of fiber-based fabrication technologies. First, the predominance of electrospinning across all highly cited patents underscores its central role as the most mature and broadly adopted platform, particularly for designing implantable and therapeutic systems that require finely tuned mechanical behavior, controlled porosity, and advanced surface functionality. The concentration of patents in cardiovascular applications highlights a strong technological focus on devices that demand conformability, biocompatibility, and integration with dynamic biological tissues. At the same time, the inclusion of nanocomposite and optoelectronic fiber systems signals a growing interdisciplinary expansion, in which electrospun materials are being engineered for multifunctional roles spanning diagnostics, sensing, responsive behavior, and hybrid biomedical–electronics interfaces. Despite these advances, the patent landscape also reveals important gaps, including limited representation of solvent-free or environmentally sustainable processes, underexplored clinical domains beyond cardiovascular and wound-care applications, and relatively few patents leveraging programmable fiber architectures or real-time bio responsive properties. These gaps highlight opportunities for future innovation, particularly in areas such as scalable green manufacturing, personalized fiber-based therapeutics, and next-generation smart biomaterials capable of dynamic interaction with their biological environment.

### 3.4. Example of Patents in Fiber-Based Fabrication Technologies for Tissue Engineering and Drug Delivery Systems

As part of this patentometric analysis, the three patents most closely aligned with the study’s objective were determined, aiming to offer insights into recent technological advancements in fiber-based fabrication methods, particularly in the context of tissue engineering and drug delivery. These patents were identified and selected using the analytical capabilities of the PatSeer platform, which employs a multifactorial relevance-ranking algorithm [36].

The first patent is CN112972777B “Electrospun PLGA/PCL fibrous membrane composite chitosan sponge scaffold for oral alveolar bone regeneration and preparation method thereof” published in 2021 and currently owned by the Affiliated Stomatological Hospital of Nanjing Medical University in China, contains a new composite scaffold with a bilayer structure made from polylactic acid-glycolic acid/polycaprolactone (PLGA/PCL) fibrous membranes and chitosan sponges for the regeneration of the oral alveolar bone. This invention addresses the challenge of balancing bone formation with soft tissue invasion. The PLGA/PCL layer acts as a selective barrier, preventing fibroblast infiltration while allowing nutrient diffusion, thereby creating an optimal environment for bone tissue regeneration. The high porosity of 91.14% to 95.74% and a pore size distribution of more than 100 μm in 94–99% of the structure enable efficient cell infiltration and nutrient exchange, making it highly suitable for promoting bone tissue regeneration at defect sites. This method yielded a thickness of the electrospun PLGA/PCL fiber membrane ranging from 150 to 250 μm, with a fiber filament diameter of 500–820 nm. The preparation method involves electrostatic spinning, polydopamine functionalization, and chemical crosslinking to ensure the tight integration of the components. The adaptability of the scaffold to irregular bone defect shapes further enhances its practical application in clinical settings, making it a significant advancement in the field of tissue engineering for oral alveolar bone regeneration.

The second most cited patent, IN480922A1 “An improved tissue engineering scaffold and a method for fabrication thereof”, was published in 2023 and is currently owned by the Indian Institute of Technology Kanpur, which relates to a new approach for the production of an improved tissue engineering scaffold. Its main objective is to supplement artificial skin that closely resembles the microenvironment of the skin’s dermal layer. A central component of this invention is a multilayer nanostructured scaffold that contains at least one layer of preseeded fibroblast cells. It is produced from electrospun polymer nanofibers, which are created by first dissolving the polymer in suitable solvents and then spinning it onto a substrate. The fibers are then coated with algal polysaccharides (especially kappa carrageenan, iota carrageenan, and lambda carrageenan) at a concentration ranging from 0.05% to 1.5% *w*/*w*. This modification significantly improved the cell adhesion and proliferation on the scaffold. Various cell types, particularly fibroblasts, can colonize the scaffold. This process enables the use of a wide range of polymers, including cellulose acetate, chitosan, and polycaprolactone. Colonization is also possible using different cell types, including fibroblasts, keratinocytes, melanocytes, stem cells, endothelial cells, osteoblasts, and osteocytes. The resulting material structurally and functionally mimics the innate extracellular matrix (ECM) of the skin, resembling the microenvironment of the dermal layer. Therefore, this development represents an advancement in the field of dermal skin substitutes and can serve as an alternative to animal testing. It also holds potential as a model for photo-aging against UV light, skin cancer research, wound healing, pharmacological interventions for drug delivery, and high-throughput screening.

The third patent is CN106913904B “Micro-nano tissue engineering scaffold with immunotherapy function and preparation method thereof,” published in 2020 and currently owned by The First Affiliated Hospital of Soochow University in China, describes a micro-nano tissue engineering scaffold that combines both immunotherapeutic and tissue-regenerative functions. The foundation of the system is a scaffold produced by electrospinning, followed by surface activation to introduce functional groups. This enables covalent grafting of specific antibodies such as IgG or CD40. This structure facilitates the localized release of antibodies, thereby contributing to the inhibition of tumors. The scaffold serves as a stable matrix for regenerating damaged tissue. Through this combination of tumor suppression and tissue regeneration, the system addresses the central weaknesses of existing immunotherapies by reducing the need for high antibody doses and minimizing potential side effects. Notably, this approach combines tumor growth suppression and tissue regeneration, making it a more effective and patient-friendly treatment option for tumor-related diseases. Such integration of therapeutic efficacy with regenerative capacity underscores the potential of nanofiber-based systems to work as multifunctional drug delivery platforms, taking it one step further. Electrospun and related fiber-based scaffolds can enable localized and sustained release of bioactive agents while simultaneously supporting tissue repair, thereby addressing critical pharmaceutical challenges such as dose optimization, stability of sensitive therapeutics, patient compliance, and the reduction in systemic side effects.

Together, these three patents illustrate how fiber-based fabrication technologies are driving the next generation of functional biomaterials by integrating structural, regenerative, and therapeutic properties within a single platform. From bone regeneration scaffolds with optimized porosity to multilayered dermal substitutes that mimic the extracellular matrix and multifunctional constructs combining immunotherapy with tissue repair, these inventions highlight the versatility of electrospinning, melt electrowriting, and Forcespinning^®^ in addressing unmet biomedical needs. Beyond their individual technical advances, they demonstrate a broader trend in which fiber-based materials are being engineered not only as passive scaffolds but as active therapeutic systems, capable of guiding cell behavior, delivering bioactive molecules, and improving clinical outcomes. This underscores the translational potential of fiber-based functional biomaterials in bridging fundamental research with practical applications in tissue engineering, drug delivery, and regenerative medicine.

### 3.5. Translational Implications for Pharmaceutics and Biomaterials

The patent trends identified in this study demonstrate the dynamic evolution of fiber-based fabrication technologies and highlight their transformative potential for pharmaceutical research and the development of functional biomaterials. The steady rise in filings until 2022 reflects the increasing maturity of electrospinning, Forcespinning^®^, and melt electrowriting. Even with the slight decline in 2023–2024, the sustained patenting activity underscores the long-term relevance of these approaches for advancing tissue engineering and drug delivery.

By enabling the design of systems with tunable properties, these technologies open new avenues for creating drug delivery platforms, scaffolds, and therapeutic devices that combine structural support with bioactive functionality. Such innovations are not only advancing pharmaceutical formulations—by improving drug solubility, bioavailability, and controlled release—but also driving the emergence of multifunctional biomaterials capable of addressing complex clinical needs in tissue engineering and regenerative medicine.

Geographic patterns further reinforce translational implications. China’s leadership, primarily driven by academic institutions such as Shanghai University, demonstrates how university-led research is fueling rapid exploration of fiber-based drug-delivery systems and regenerative medicine applications. Meanwhile, contributions from Europe and North America reveal a globally competitive landscape, where regional strengths in medical technology and materials science are being translated into pharmaceutical and biomaterials applications through interdisciplinary collaboration.

Finally, sectoral and IPC code analyses show that innovations at the intersection of medical technology, textiles, and chemical processing are directly influencing pharmaceutical formulation. Current research trends reveal a sustained focus on exploiting the unique physical and chemical versatility of electrospinning to engineer nanofiber systems with high drug-loading capacity, enhanced dissolution of poorly soluble compounds, and precisely tunable release kinetics [27]. Moreover, electrospun and melt electrowritten fibers can enable the encapsulation of sensitive biomolecules, such as proteins, peptides, and nucleic acids, thereby enhancing their stability and therapeutic efficacy. Increasingly, investigations center on optimizing solution rheology, polymer chain entanglement, conductivity, and solvent volatility—key physicochemical parameters that dictate jet stability, fiber diameter distribution, and internal microstructure [55]. These variables, together with post-spinning chemical modification strategies, enable precise control over surface chemistry, wettability, degradation kinetics, and molecular interactions within the fiber matrix. Such control is essential for the effective encapsulation and stabilization of sensitive biomolecules including proteins, peptides, and nucleic acids, thereby expanding electrospinning’s relevance in advanced pharmaceutics and regenerative medicine [29,56]. Collectively, these advances address key challenges in pharmaceutics, including poor bioavailability [11,57,58], premature degradation, and the need for patient-tailored delivery strategies, positioning fiber-based fabrication as a cornerstone of next-generation drug delivery systems and biomaterial-based therapeutics design [59].

Building on these physicochemical and biomolecular advances, research efforts are directed toward expanding the functional and translational potential of the electrospinning technology. Current research increasingly emphasizes the development of adaptive, multifunctional fiber systems by incorporating stimuli-responsive polymers, bioactive nanocomposites, and hybrid biomaterials that enable dynamic interactions with the biological environment. Simultaneously, significant progress is being made in refining process control, leveraging real-time monitoring, data-driven optimization, and closed-loop regulation to achieve higher reproducibility and clinically scalable manufacturing. These trends are complemented by a growing shift toward environmentally responsible, regulatory-compliant processing, including the adoption of aqueous or solvent-free electrospinning routes. This behavior exhibits a shift toward electrospun biomaterials that are not only structurally sophisticated but also engineered for practical deployment in advanced therapeutics, diagnostics, and regenerative interventions.

The prevalence of electrospinning and related methods in patent activity underscores their significant potential to develop nanofiber-based platforms capable of delivering a broad spectrum of therapeutics, from small molecules to complex biologics. These platforms exemplify the role of functional biomaterials, integrating structural, mechanical, and biochemical properties that enable dynamic interactions with biological systems and enhance therapeutic outcomes.

Beyond this functional versatility, it is also essential to consider the types of biologically active compounds incorporated into micro- and nanofibers across these fabrication methods. Electrospinning, Forcespinning^®^, and melt electrowriting have all been used to encapsulate a broad range of active pharmaceutical ingredients (APIs), including small-molecule drugs (e.g., antibiotics such as tetracycline, ciprofloxacin) [29,50], nonsteroidal anti-inflammatory agents such as diclofenac, anti-inflammatory agents like diclofenac, anticancer drugs such as paclitaxel and doxorubicin) [29], as well as biologics (e.g., growth factors, cytokines, peptides, vaccines, and nucleic acids) [38,55]. These APIs typically fall within pharmacological classes that address major therapeutic challenges: antimicrobial agents for infection control, nonsteroidal anti-inflammatory drugs for pain and inflammation, chemotherapeutics for localized cancer therapy, and biomolecules for regenerative medicine and gene modulation. Their incorporation into fibers demonstrates the advantages of fiber-based platforms for improving drug solubility, enhancing bioavailability, protecting labile compounds from degradation, and enabling spatial and temporal control of release. The patent landscape indicates increasing interest in multifunctional delivery systems capable of sequential release, stimuli-responsiveness, or integration with diagnostic elements which are features well supported by nanofiber architectures.

Despite the potential that these fiber-based technologies offer in a variety of fields, they also encounter several limitations. Electrospinning, though the most widely adopted, is particularly sensitive to solution rheology, conductivity, polymer chain entanglement, solvent volatility, and environmental parameters, all of which influence jet stability and fiber morphology. Its low production rate and frequent reliance on organic solvents present additional challenges for scalability and environmental sustainability [14]. Conversely, melt electrowriting offers higher precision without solvents, and Forcespinning^®^ provides higher throughput, but both remain less explored in biomedical applications. The need for enhanced process control, greener fabrication methods, and standardized quality metrics remains evident across all three technologies.

On the other hand, although there is ongoing progress, substantial research gaps remain at the interface of material design, process optimization, and translational application. There is a lack of systematic, comparative analyses that integrate technological performance with biomedical outcomes across electrospinning, Forcespinning^®^, and melt electrowriting. Critical questions persist regarding how fabrication parameters influence the stability of sensitive APIs, how fiber architectures correlate with in vivo performance, and which process refinements are required to achieve reproducible, clinically scalable manufacturing [38,51]. These concerns echo the perspectives of Mieszczanek et al., Cao et al., and Wang Y et al., who emphasize the need for improved accuracy, consistency, and automation [47,50,60]. Moreover, the field still lacks unified standards for evaluating fiber quality, drug loading efficiency, release kinetics, and long-term biocompatibility—factors essential for regulatory approval and industrial adoption [29,52,60]. Addressing these gaps will be crucial to transforming emerging fiber-based fabrication technologies into robust, clinically relevant platforms that advance the next generation of therapeutic and regenerative solutions.

## 4. Conclusions

This study provides a comprehensive overview of the global technological landscape of three fiber-based fabrication technologies: electrospinning, Forcespinning^®^, and melt electrowriting within the context of tissue engineering, functional biomaterials and drug delivery systems between 2020 and 2024. This study was conducted in response to the rapid growth of these fields and addresses the lack of an integrated patentometric analysis that examines all three technologies within a biomedical framework. By applying a Competitive Technology Intelligence (CTI) methodology and analyzing Extended Patent Families (EFAMs), the study offers a non-redundant, global perspective on the technological evolution and application of these fiber-based techniques. This approach not only characterizes innovation trends and key actors but also highlights how these techniques are supporting the development of functional biomaterials with direct implications for advanced drug delivery systems and regenerative medicine. To facilitate the understanding of their evolving technological landscape, and offer insights that can support translational applications, anticipate competitive trends, and guide R&D strategies.

A total of 3557 EFAMs were identified, revealing a strong geographic concentration of patenting activity in Asia, particularly China. Shanghai University stands out as the leading institutional contributor, underscoring the influence of academic organizations supported by national strategies that promote innovation and intellectual property development. The principal industrial sectors associated with these technologies correspond to medical and dental technology, special-purpose machinery, and specialized textile production. IPC classifications indicate that nanofibers, functionalized materials, and bioactive composites remain central to invention strategies. Electrospinning (IPC D04H1/728) emerges as the most dominant technology, reflected in both filing volume and citation activity. While Forcespinning^®^ and melt electrowriting continue to expand, their overall patent presence and impact remain comparatively lower, although adoption trends suggest growing relevance in the coming years.

While the CTI framework enables the identification of major trends, jurisdictions, and stakeholders, the study’s conclusions are bounded by the limitations of patent data, including cross-country variability in documentation, classification, and disclosure practices. Patent analysis alone cannot fully capture technological readiness levels or commercialization routes, nor does the 2020–2024 timeframe fully encompass long-term trajectories. The exclusive reliance on patent-derived information excludes complementary insights from scientific publications and industrial reports, which could provide a broader perspective on emerging research directions and non-patented innovations. Moreover, patent analysis alone cannot fully resolve questions related to clinical performance, material properties, or translational viability. The findings should be interpreted as indicators of innovation and technological direction rather than comprehensive evaluations of efficacy or safety. Future work should integrate patent data with experimental, regulatory, and manufacturing analyses to build a more complete picture of the field.

Overall, the patent trends identified here highlight the dynamism of fiber-based fabrication technologies and outline a roadmap for future innovation. As these platforms advance toward greater precision, multifunctionality, and translational impact, they are poised to play a central role in next-generation pharmaceutical formulation and biomaterial-based therapeutic delivery. The insights provided in this study offer valuable guidance for researchers, industry stakeholders, and policymakers seeking to align strategic R&D decisions with evolving technological opportunities.

## Figures and Tables

**Table 1:** Top five most cited patents published from 2020 to 2024 on electrospinning, melt electrowriting, and Forcespinning^®^ technologies for tissue engineering and drug delivery. Citation data retrieved on 21 April 2025. Note: Patents were obtained from the Patseer platform, which can access 172 M+ Publications; 108+ Authorities; 134.7 M+ Full-text records; 74 M+ Families; 63M + 97 M+ Simple family and 94 M+ Extended family [30].

Current Owner	Year	Key Invention	Citations
**1. US10849754B2 Heart valve sealing devices and delivery devices therefor.**			
Edward Lifesciences Corporation (Irvine, CA, USA)	2020	Minimally invasive device for native valve regurgitation treatment. Coaption element improves leaflet closure during systole by filling the space between the valve leaflets. Its symmetrical geometry and hourglass-shaped proximal ensure anatomical conformity. Paddle system actuated via a shaft-cap mechanism enables movement between open and closed positions to engage valve leaflets. Flexible mesh paddles mounted on a 3D extension member for leaflet adaptation. Features teardrop-shaped gaps between paddles and coaption element.	113
**2. US11013600B2 Covered prosthetic heart valve.**			
Edward Lifesciences Corporation (Irvine, CA, USA)	2021	Prosthetic heart valve with a radially collapsible and expandable frame, including inflow and outflow ends, and an integrated leaflet structure. Multi-zone covering with a floating portion that accommodates radial deformation and supports dynamic interaction with cardiac motion. The floating portion positioned between fixed woven zones minimize friction and reduce tissue abrasion. Circumferentially spaced openings aligned with frame struts support fluid dynamics and promoted anatomical integration. The protective folded textile layers and helically wrapped reinforcements enhance mechanical stability and reduce abrasion risks.	109
**3. US10822542B2 Perovskite/polymer composite luminescent material, preparation method and use.**			
Beijing Institute of Technology and Zhijing Nanotech Co (Beijing, China).	2020	In situ formation of perovskite nanoparticles within polymer matrices via solution-based processing that enables direct integration without post-synthesis purification, maintaining nanoparticle dispersion and optical performance. The surface functionalization of perovskite nanoparticles with organic ligands contributes to improved optical quality via defect passivation. Electrospinning and other solution-based deposition techniques (e.g., spin coating, dip coating, spray coating) enable fabrication of flexible, uniform composite nanofibers with homogeneously dispersed luminescent nanoparticles. Surface ligands facilitate nanoparticle dispersion and prevent aggregation. Scalable processing via solution-based techniques (e.g., electrospinning, spin coating) supports industrial application in flexible optoelectronics.	66
**4. US10993809B2 Heart valve sealing devices and delivery devices therefor.**			
Edward Lifesciences Corporation (Irvine, CA, USA)	2021	Valve repair device for native valve insufficiencies via minimally invasive transcatheter access. Features a single woven strip comprising a thinner central portion and more rigid edge. Includes a coaption element and adjustable paddles, configured to switch between open and closed positions. The paddles are attached to valve leaflets, enabling coaptation and closure. The device is deliverable through a transcatheter system for minimally invasive, non-open-heart procedures. Its folded design allows compact configuration for percutaneous deployment.	58
**5. US11167058B2 Hemostasis of wound having high pressure blood flow.**			
Virginia Commonwealth University Richmond, VA, USA)	2021	This invention uses clay minerals to promote hemostasis. Electrospun delivery systems containing kaolin provide a flexible and high-surface-area matrix suited for irregular wound profiles. No exothermic reactions upon application, preventing tissue damage. Effective for both external and internal wounds, including high-pressure arterial bleeding. Available in various physical forms (e.g., powders, granules, fibers, bandages) for different applications. Can be combined with other hemostatic agents such as fibrinogen, thrombin, and chitosan, either sequentially or simultaneously. Clay minerals such as kaolin promote blood clotting by facilitating hemostatic activity upon contact with blood.	51

## Data Availability

No new data were created or analyzed in this study. Data sharing is not applicable to this article.
